# Considerations for animal models of blast-related traumatic brain injury and chronic traumatic encephalopathy

**DOI:** 10.1186/s13195-014-0064-3

**Published:** 2014-09-05

**Authors:** Lee E Goldstein, Ann C McKee, Patric K Stanton

**Affiliations:** 1 Boston University School of Medicine and College of Engineering, 670 Albany Street, 4th Floor, Boston 02118, MA, USA; 2 Boston University Alzheimer’s Disease Center, Boston University School of Medicine, Robinson Hall, 7th Floor, Boston 02118, MA, USA; 3 US Department of Veterans Affairs, VA Boston Healthcare System, 150 South Huntington Avenue, Boston 02130, MA, USA; 4 Departments of Neurology, Cell Biology & Anatomy, New York Medical College, Basic Science Building, Rm 217, Valhalla 10595, NY, USA

## Abstract

The association of military blast exposure and brain injury was first appreciated in World War I as commotio cerebri, and later as shell shock. Similar injuries sustained in modern military conflicts are now classified as mild traumatic brain injury (TBI). Recent research has yielded new insights into the mechanisms by which blast exposure leads to acute brain injury and chronic sequelae, including postconcussive syndrome, post-traumatic stress disorder, post-traumatic headache, and chronic traumatic encephalopathy, a tau protein neurodegenerative disease. Impediments to delivery of effective medical care for individuals affected by blast-related TBI include: poor insight into the heterogeneity of neurological insults induced by blast exposure; limited understanding of the mechanisms by which blast exposure injures the brain and triggers sequelae; failure to appreciate interactive injuries that affect frontal lobe function, pituitary regulation, and neurovegetative homeostasis; unknown influence of genetic risk factors, prior trauma, and comorbidities; absence of validated diagnostic criteria and clinical nosology that differentiate clinical endophenotypes; and lack of empirical evidence to guide medical management and therapeutic intervention. While clinicopathological analysis can provide evidence of correlative association, experimental use of animal models remains the primary tool for establishing causal mechanisms of disease. However, the TBI field is confronted by a welter of animal models with varying clinical relevance, thereby impeding scientific coherence and hindering translational progress. Animal models of blast TBI will be far more translationally useful if experimental emphasis focuses on accurate reproduction of clinically relevant endpoints (output) rather than scaled replication of idealized blast shockwaves (input). The utility of an animal model is dependent on the degree to which the model recapitulates pathophysiological mechanisms, neuropathological features, and neurological sequelae observed in the corresponding human disorder. Understanding the purpose of an animal model and the criteria by which experimental results derived from the model are validated are critical components for useful animal modeling. Animal models that reliably demonstrate clinically relevant endpoints will expedite development of new treatments, diagnostics, preventive measures, and rehabilitative strategies for individuals affected by blast TBI and its aftermath.

## Introduction

Traumatic brain injury (TBI) resulting from blast exposure affects combatants and civilians around the world [1]-[3]. Recent estimates indicate that 10 to 20% of the 2.5 million US military service members deployed to Iraq and Afghanistan may be affected by TBI and the majority of these injuries are associated with blast exposure [4]-[13]. Individuals exposed to blast are at increased risk of acute neurological deficits, persistent pathological changes in the brain, and chronic neuropsychiatric and cognitive disability [1],[10]-[24]. Blast exposure is a known precipitant of brain injury in animals [22],[25]-[40] and humans [14],[19]-[22],[41]-[43], including individuals with repeated exposure to low-level blast [23],[24]. Recent research has uncovered neuropathological and mechanistic connections between blast exposure and chronic traumatic encephalopathy (CTE), a progressive tau protein neurodegenerative disease documented in athletes with repetitive concussive and subconcussive head injury [44],[45] and in military veterans with history of blast exposure [21],[22]. Recent experimental studies have demonstrated TBI-linked and CTE-linked tau neuropathology and neurobehavioral deficits in laboratory animals following blast exposure [22],[39]. These findings suggest a mechanistically causal connection between blast exposure and organic brain injury. Collectively, these findings represent a major paradigm shift in medical understanding of acute and chronic effects of blast exposure on brain structure and function.

Growing awareness of the long-term consequences of blast TBI and the large number of returning military service members and civilians who have experienced blast exposure necessitate increased research to better understand, diagnose, and treat acute and chronic effects of blast-related neurotrauma. Research advances have yielded fundamental insights into the neurobiological basis, biomechanical determinants, and pathophysiological mechanisms by which blast exposure induces acute brain injury and chronic neurological sequelae [22],[39]. The field is now poised for translational research to develop new diagnostics, treatments, preventive measures, and rehabilitative strategies for individuals affected by blast neurotrauma. These efforts will be facilitated by critical assessment of unresolved clinical and translational issues that currently impede progress on both fronts.

Translational research in this area has been hampered by a number of methodological issues, including lack of consensus regarding what constitutes an appropriate animal model of blast TBI and how to evaluate the validity of experimental results obtained from these models. To be useful, animal models must have a well defined purpose and recapitulate clinically relevant features – including neuropathological hallmarks, neurophysiological defects, neurobehavioral deficits, and cognitive impairments – that correspond to abnormalities observed in humans exposed to blast. Animal models that accurately recapitulate human pathology are a critical prerequisite for understanding pathogenic mechanisms and developing new diagnostics and therapeutics for TBI and CTE [46]. Determining the extent to which common neurophysiological mechanisms eliciting TBI are shared by differing types and severity of incident traumas (for example, blast, impact, polytrauma), and how these mechanisms contribute to the temporal course and clinical evolution (for example, persistence, progression, resolution) of the resulting injuries, will be key factors for successful translational efforts in this arena. This review will address key issues for animal models of blast-related TBI and CTE.

## Clinical considerations

### Pathobiology of blast traumatic brain injury, chronic sequelae, and comorbidities

Emerging clinical evidence indicates that repetitive head injury in contact-sport athletes and blast exposure in military service personnel may be associated with long-term neuropathology, psychiatric disturbances, endocrine abnormalities, and cognitive impairment in susceptible individuals [1]-[3],[15]-[17],[19],[22],[44],[45],[47]-[52]. McKee and colleagues recently reported a large case series of postmortem brains from a cohort of 85 subjects with histories of mild TBI in which evidence of CTE neuropathology was detected in 68 subjects (mean age, 59.5 years), including 21 military veterans who served in World War II, Korea, Vietnam, Gulf War, Iraq or Afghanistan [44]. Postmortem brains from 18 age-matched and sex-matched subjects without a history of TBI did not demonstrate neuropathological evidence of CTE. A controlled case series [22] and a single case report [21] found evidence of CTE neuropathology in postmortem brains from a total of five military veterans with documented histories of blast exposure during deployment to Iraq or Afghanistan, including one veteran with single blast exposure and no documented history of antecedent, coincident, or subsequent TBI [22]. Notably, four of the five blast-exposed military veterans with CTE neuropathology also carried a clinical diagnosis of post-traumatic stress disorder (PTSD) [22]. A critical observation from these studies was that CTE pathology documented in the brains of blast-exposed military veterans was neuropathologically indistinguishable from CTE pathology observed in the brains of athletes with a history of repetitive concussive or subconcussive head injury [22]. Neuropathological hallmarks in both groups included widespread cortical foci of perivascular tau pathology, microgliosis, astrocytosis, myelinated axonopathy, and focal neurodegeneration. Clinical symptoms in both groups were also similar and included progressive affective lability, irritability, distractability, executive dysfunction, memory disturbances, and cognitive deficits consistent with CTE [44],[45].

A recent study we conducted in a blast neurotrauma mouse model implicates blast wind-induced acceleration–deceleration of the head (bobblehead effect) as a primary pathogenic mechanism by which blast exposure induces brain injury. Neuropathological hallmarks of blast neurotrauma in humans and our mouse model include myelinated axonopathy, focal microvasculopathy, chronic neuroinflammation, frank neurodegeneration, and phosphorylated tau proteinopathy [22]. Critically important in this study are experimental findings obtained from a blast neurotrauma mouse model that recapitulated core neuropathological and neurobehavioral features of blast-related TBI and CTE in humans. The results of this study identified common pathogenic determinants leading to CTE in blast-exposed military veterans and head-injured athletes [22]. This same study provided mechanistic evidence that supports this association by showing that laboratory mice exposed to experimental blast also develop TBI-linked and CTE-linked neuropathology [22],[39].

**Figure 1:**
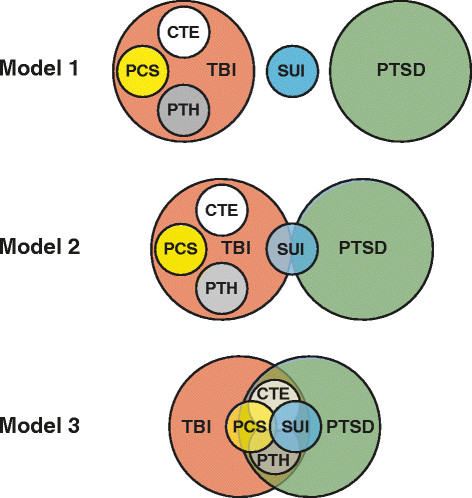
**Conceptual models for blast-related neuropsychiatric disorders.** Traumatic brain injury (TBI) is presumed to be a requisite antecedent to post-concussive syndrome (PCS), chronic traumatic encephalopathy (CTE), and post-traumatic headache (PTH). Post-traumatic stress disorder (PTSD) is characterized as an independent clinical entity in Models 1 and 2, but not in Model 3. Suicide-related ideation and behaviors (SUI) subsumes a constellation of psychiatric attributes (for example, self-directed violence, suicidal ideation and intent, attempted suicide, completed suicide) that may segregate as independent clinical entities. Note that these conceptual models do not distinguish mediating (causal) and confounding (noncausal) interactions. In Model 1, the clinical entities are maximally independent. In Model 2, the clinical entities incompletely overlap. In Model 3, the clinical entities are maximally interdependent. Modulating factors (age, sex, trauma history, genotype), comorbidities (for example, frontal lobe syndrome, major affective disorders, substance use disorders), and situational factors (for example, psychosocial stress, affect flooding, firearm availability) may complicate diagnostic differentiation and clinical management. Given the diagnostic uncertainty and clinical complexity of blast TBI-related neuropsychiatric conditions, a one-size-fits-all management strategy for each clinical disorder is unlikely to be effective.

Although overlapping clinical features of blast TBI and related sequelae suggest common pathobiology, the neurobiological substrates and pathophysiological mechanisms underpinning these clinical entities and interactions are poorly understood. Moreover, the temporal course of secondary responses in the peri-acute and chronic stages following acute injury is largely unknown. Recently, a number of blue ribbon committees have reviewed the available clinical evidence and established consensus definitions with respect to diagnostic criteria and management guidelines for TBI, concussion, and related conditions [53]-[58]. While these efforts have brought some order to the field, the reality is that diagnosis, prognosis, triage, and monitoring for these conditions remain grounded on clinical criteria with unproven relationships to underlying pathophysiology. As a consequence, clinical nosology and pathophysiological understanding are uncoupled. This situation impedes optimal delivery of medical care for affected individuals and stymies efforts to implement personalized medical management and effective therapeutic intervention for TBI and related sequelae. What little is known of TBI pathobiology suggests that optimal clinical care requires consideration of interacting pathologies that affect different brain regions and lead to different symptoms (Figure 1).

Recent evidence linking blast exposure to development of CTE [21],[22],[44] underscores the importance of considering the highly heterogeneous nature of the inciting traumatic injuries and the complex pathobiological interactions that arise in the setting of mild TBI [59]. CTE is a slowly progressive tau protein-linked neurodegenerative disease associated with sports-related concussive and subconcussive head injury as well as military-related blast exposure [21],[22],[44]. While phosphorylated tau protein neuropathology is a prominent component of CTE, associated features underscore the importance of other disease-linked pathologies, including: focal microvasculopathy, perivascular neuroinflammation, and myelinated axonopathy; a distinctive pathological distribution pattern (for example, predilection for sulcal depths, focal perivascular epicenters) that implicates biomechanical injury at sites of shear stress concentration; involvement of other CTE-associated pathogenic proteins (for example, TAR DNA-binding protein 43); and a characteristic pathological progression that correlates with clinical symptoms [44]. Converging evidence suggests that tau proteinopathies may involve prion-like propagation in the brain [60]-[62]; however, this mechanism remains speculative with respect to disease progression in CTE.

The clinical presentation in CTE includes attentional and concentration disturbances, short-term memory problems, impulsivity, irritability, chronic headache, explosive aggression, sleep and mood disturbances, psychosocial impairments, and suicidality [44],[45],[63]. Clinical features of CTE overlap significantly with PTSD [64],[65], a profoundly disabling neuropsychiatric disorder that is often associated with blast TBI.

The clinical courses of TBI, CTE, and, to some extent, PTSD are variable, frequently overlap clinically, and often include neuropsychiatric signs and symptoms attributable to organic brain syndromes that affect the frontal lobes. Since the frontal lobes are anatomically vulnerable to traumatic injury, it is not surprising that TBI-related neuropathology often involves this brain region. Characteristic disturbances of frontal lobe function include impairments in working memory, planning, multitasking, complex decision-making, judgment, empathy, and executive function [66],[67]. Changes in personality and social behavior may be prominent. Other notable signs of frontal lobe dysfunction are impulsivity, emotional lability, and disinhibition. Global damage to frontal lobe function is characteristically marked by an inability to inhibit stimulus-initiated behavioral programs [66],[67]. Neurological derangements involving the frontal lobes can thus result in release of inappropriate, impulsive, or maladaptive behaviors that would otherwise be inhibited. Persistent frontal lobe dysfunction following blast TBI may result in failure to extinguish responses to trauma-related stimuli and maintenance of stimulus–response linkages. Blast-related TBI is commonly associated with PTSD, and in this setting frontal lobe dysfunction may result in symptom persistence, resistance to cognitive behavioral interventions, and increased risk of maladaptive, impulsive, and injurious behaviors. In the military context, frontal lobe-mediated neuropsychiatric disturbances may compromise operational judgment, tactical performance, personnel safety, and mission objectives.

Individuals affected by blast TBI present many challenges with respect to clinical management, longitudinal monitoring, and risk assessment. In addition, therapeutic responses in this population may be highly variable as a consequence of the heterogeneous nature of the inciting traumas, affected neuroanatomical substrates, and underlying pathobiological responses. Patients with confirmed or suspected organic brain syndromes involving the frontal lobes present additional challenges. For these reasons, a one-size-fits-all management approach for blast TBI is unlikely to be effective. These considerations also underscore the importance of pathobiologically informed diagnostic classifications, subtype identification based on affected neurobiological systems, and differential diagnoses that include common comorbidities (for example, substance use disorders, major affective disorders, PTSD). The need for longitudinal care and clinical vigilance in this setting is paramount.

### Pathobiologically informed diagnostic criteria and biomarkers

Microscopic structural changes in the brain that characterize blast TBI and related sequelae cannot be identified during life using currently available diagnostic tools. A number of candidate biomarkers of mild TBI have been identified in blood and cerebrospinal fluid, including S100-B, myelin basic protein, neuron-specific enolase, glial fibrillary acidic protein, ubiquitin carboxy-terminal hydrolase L1, spectrin breakdown products, microtubule-associated tau protein, secreted forms of the amyloid precursor protein (sAPP-α, sAPP-β), γ-enolase, neurofilament polypeptide species, various interleukins and cytokines, and small noncoding RNA molecules (reviewed in [3],[68]-[75]). A variety of neuroimaging markers have been identified and proposed as diagnostic biomarkers of mild TBI [48],[76],[77].

Despite these advances, the pathobiology of blast-related TBI remains poorly understood, thus impeding clinical validation of TBI-specific biomarkers and risk factors. These gaps compromise diagnostic evaluation, endophenotype differentiation, clinicopathological correlation, and epidemiological characterization. In addition, the absence of validated biomarkers for mild TBI undermines screening, prognosis, staging, risk assessment, and longitudinal monitoring. Moreover, different biomarkers may be needed to detect and differentiate TBI persistence, progression, and conversion to chronic sequelae, including CTE. The urgent need for diagnostic biomarkers extends to commonly associated comorbidities such as PTSD. Validated biomarkers are also needed for clinical trial evaluation of emerging therapeutics. Cohort stratification, therapeutic efficacy, and regulatory clearance critically depend on clinical biomarker sensitivity, specificity, and utility. Indeed, biomarker availability poses a ‘go–no go’ challenge for development of effective treatments. In this regard, the logic and strategy for biomarker assessment in Alzheimer’s disease is instructive [78].

### Genetic risk factors and modulatory influences

Genetic factors, epigenetic changes, environmental exposures, trauma history, interaction with comorbid conditions, and other modulatory factors (for example, age, sex, medical history, environmental exposures, lifestyle and nutritional factors) collectively comprise an individual’s risk profile for disease expression and progression. Identification of major risk and modulatory factors is essential for understanding disease risk, initiation, progression, and outcome. Genetic markers of increased vulnerability to neurotrauma are emerging and numerous studies implicate apolipoprotein E4 as a major genetic risk factor associated with poor clinical outcome after TBI (reviewed in [79]-[83]). Analysis of other genetic risk factors, epigenetic markers, and other modulatory influences will require validated clinical and molecular phenotyping based on disease-linked attributes and mechanistic interpretation based on detailed understanding of the underlying pathobiology.

### Need for effective treatments and preventive measures

**Figure 2:**
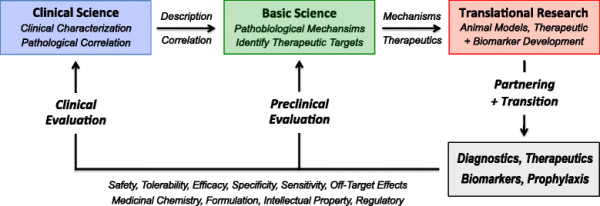
**Translational research pathways and relationship to basic and clinical sciences.** Rational development and clinical implementation of safe and effective diagnostics and therapeutics for blast traumatic brain injury, related sequelae, and associated comorbidities will require detailed mechanistic understanding of the underlying pathology (clinical science) and pathogenic mechanisms (basic science) across all stages of the disease (initiation, progression, interaction, termination). A strong clinical and basic science foundation is an essential prerequisite for efficient identification and validation of new diagnostic biomarkers and therapeutic targets. Multidisciplinary translational research teams are essential for these efforts. Collaborative partnerships with biotechnology and pharmaceutical companies can provide additional resources, share development risk, and – under favorable conditions – shoulder transition costs, including product development, intellectual property and regulatory management, safety and toxicological testing, preclinical efficacy evaluation, and clinical trial testing.

Optimal clinical care for individuals affected by blast TBI and related conditions is dependent on availability of safe and effective therapy. Unfortunately, there are no currently available US Food and Drug Administration-approved treatments to halt, slow, reverse, or correct the underlying pathobiology. Direct and indirect costs associated with this therapy gap for military-related TBI, sequelae, and comorbidities, although unknown, are undoubtedly large and likely to increase over time given the types of injuries and numbers of affected service members and veterans [84],[85]. A major factor contributing to the absence of effective treatments for TBI-related conditions is attributable to the limited knowledge and heterogeneous nature of pathogenic mechanisms underpinning TBI-related injuries, secondary responses, chronic sequelae, and associated comorbidities. Rational therapeutic development and efficient clinical delivery of effective therapeutic and prophylactic interventions for TBI will be greatly facilitated by robust multidisciplinary translational research focused on identifying pathogenic mechanisms, therapeutic targets, diagnostic biomarkers, and pathobiology-modifying interventions (Figure 2).

As for many multifactorial disorders with complex etiologies, a clinical armamentarium for TBI is expected to include a variety of small-molecule pharmaceuticals that target distinct pathways altered by TBI as well as adjunctive treatments, including nutraceuticals, electroceuticals, cognitive behavioral therapies, computer-assisted technologies, and other therapeutic modalities. Our recent observations that blast exposure can produce persistent impairments in synaptic plasticity essential for learning and memory [21] support the idea that bioavailable small-molecule therapeutics that modulate neurotransmission and synaptic plasticity should be considered high-priority targets for TBI-related conditions. Similarly, the potential for some overlap in neuropathology associated with TBI and neurodegenerative diseases further supports the idea of developing adjunctive therapeutics with synergistic cognitive enhancing effects. Regardless of the targets, one point is clear: development of effective interventions for TBI and related conditions is unlikely to be successful without further progress in understanding the substrate pathobiology.

## Translational considerations

### Multidisciplinary translational research is essential for clinical progress

Clinical delivery of effective diagnostics, therapeutics, preventive measures, and rehabilitative strategies for TBI-related indications will require substantial investment to support multidisciplinary translational research teams with expertise, technical resources, and infrastructure that span various clinical specialties (for example, neurology, neurosurgery, psychiatry, pathology), basic and applied sciences (for example, neurobiology, genetics, physiology, pharmacology, psychology, medicinal chemistry), and engineering disciplines (for example, mechanical, biomedical, bioinformatics). A new cadre of investigators and multidisciplinary collaborations is needed to advance translational progress, stimulate transformative innovation, and accelerate development of safe and effective clinical products for TBI and related conditions. A well-resourced national initiative is urgently needed to attract, motivate, support, and sustain the nation’s best and brightest to enter the field, evaluate high-risk/high-reward hypotheses, and translate scientific advances into clinically useful diagnostics, therapeutics, preventive measures, and rehabilitative strategies. A successful translational strategy will probably include bio-innovators with proven track records of organizing translationally oriented research teams that successfully tackle challenging biomedical problems with innovative solutions. Paradigm-shifting advances are likely to be made by research teams that are capable of working across boundaries between established disciplines and willing to consider outsider perspectives [86]. An environment that fosters truly transformative innovation will require focus on long-term objectives, incentives for risk-taking, tolerance for failure, and merit-based evaluation that prioritizes disjunctive innovation and translational success over incremental progress.

### Translational considerations for blast traumatic brain injury animal modeling

Elucidating pathobiological mechanisms and identification of therapeutic targets based on pathophysiological understanding are *sine qua non* for translational development of new clinical armamentarium for TBI-related indications. Many fundamental issues related to blast neurotrauma are just beginning to be explored, including blast effects on neuronal function and physiology, microvascular structure and function, blood–brain barrier integrity, neuroinflammation and secondary responses, and brain network dynamics. Recently recognized connections between blast neurotrauma and persistent functional axonopathy and brain proteinopathy involving the microtubule-associated tau protein have only recently been recognized and require intensive investigation [22],[39]. The biological underpinnings of variabilities in clinical presentation, temporal course, long-term outcome, and individual susceptibility are poorly understood. Prognostic assessment in this setting is largely a matter of guesswork. The current situation reflects large knowledge gaps in understanding the pathobiology of acute blast neurotrauma and its aftermath. These knowledge gaps can be addressed by translational research that utilizes animal models of blast neurotrauma.

Animal models are essential tools in the translational research armamentarium. Experimental use of animal models enables hypothesis testing under controlled conditions that are often not possible in clinical research. In fact, many clinically relevant neurophysiological parameters can only be assessed in the context of animal experiments. The benefits of human TBI research in terms of relevance is often counterbalanced by technical limitations, ethical challenges, and study confounds, including limited cohort size and inclusion of subjects with heterogeneous injuries and multiple etiologies, diverse trauma histories, complicating comorbidities, and diverse risk factor profiles [59]. Moreover, clinicopathological correlation analysis, a core component of experimental studies involving animals, is often difficult or impossible in human subject research. For these reasons, translational research utilizing animal models is an essential requirement for advancing medical understanding and clinical progress.

Fundamental to animal modeling is the definition of purpose [87],[88]. Much debate in the field has centered on standardizing animal models and experimental protocols. In fact, a strong argument can be made that standardization will constrain rather than facilitate translational progress. Rather than standardization, what is needed is clarity regarding the purpose of particular models and protocols (‘what is the model modeling?’) and the validity of results and interpretations that flow from particular animal models and experimental protocols (’what do the experimental results mean?’). The number of different animal models is irrelevant. However, the interpretation and validity of experimental results derived from particular animal models are matters of great significance.

Animal models are developed and deployed for a variety of purposes, including: identification of biological substrates and pathogenic mechanisms; elucidation and validation of therapeutic targets and diagnostic markers; assessment of risks associated with new interventions; and translation of preclinical insights into clinical practice. The purpose of any particular animal model is important in so far as this purpose, declared or implied, constrains the generality and explanatory power of the answers provided by the model [87],[88]. Moreover, the interpretability of data generated by use of a particular model comprises a major criterion by which experimental and interpretive validity is established. Important in this context is the fact that validation is not conferred on an animal model *per se*, but rather on the quality and interpretation of experimental data arising from a model and on the experimental concordance of the model with clinical features and pathobiology of the human disorder that is being modeled [87]-[90]. The designation of a validated animal model thus has no inherent meaning independent of the context in which the animal model and derivative experimental results are evaluated.

These concepts are important to consider in the context of animal modeling relevant to TBI research. For example, an animal model of blast neurotrauma that emphasizes precise scaling of Friedlander blast characteristics but does not replicate brain pathology and neurobehavioral deficits corresponding to neuropathological and clinical features observed in blast-exposed humans may prove to be an ideal experimental system for ballistics investigations, but provides only limited information of clinical relevance with respect to blast TBI in humans. Conversely, an animal model of blast neurotrauma that reliably recapitulates neuropathology and neurobehavioral deficits observed in blast-exposed humans may prove useful in providing clinically relevant information regarding blast TBI, but less informative with respect to accurate determination of scaling factors and biomechanical constants for finite element analysis. A caveat is required in experimental circumstances in which deployment of a pathobiologically informed animal model provides superior mechanistic insights (that is, construct validity) and explanatory generalizability (that is, external validity), such that derivative biomechanical analyses are actually more rather than less clinically relevant. This is the case in recent studies that have combined military-relevant blast exposure conditions with animal models that exhibit clinically relevant acute and chronic blast-related neuropathology and functional sequelae [22],[39].

These considerations also apply to evaluating the validity of experimental results that use animal models. For example, the validity and relevance of experimental studies that use blast TBI animal models are frequently challenged on the basis of interspecies scaling factors and other methodological technicalities. Such arguments represent category errors, and as such are logically flawed. While technical concerns may warrant debate, these criteria are inappropriate for evaluation of experimental validity. As noted above, validation is not conferred or rejected based on the animal model *per se*, but rather on the quality and interpretation of experimental data derived from a particular animal model and applicability to mechanistic underpinnings of human TBI. Simply stated, the validity of experiments using any animal model stands or falls on the cumulative merits of the scientific results evaluated according to established criteria. By convention, validation criteria include: internal validity (reproducibility, replication); face validity (correspondence to clinicopathological features in humans); construct validity (fitness to known or suspected pathobiology); and, most importantly, predictive value and generalizability (empirical extrapolation and validation under different conditions, and ideally across species) [87]-[90]. Interspecies scaling factors and other methodological matters may be important for biomechanical calculations, but do not figure in assessing the validity of animal experiments designed to investigate the pathobiology of blast-related neurotrauma and chronic neurological sequelae.

### Reframing experimental focus on brain rather than blast

Evaluation of experimental results obtained from studies that utilize animal models of blast TBI are obligated to consider the degree to which model concordance reflects blast-related abnormalities in humans. Clinical relevance and translational utility demand that animal models of blast neurotrauma emphasize experimental fidelity of the resulting brain injury in the context of the inciting traumatic insult. Insistence on animal models that require accurately scaled versions of military-relevant blast is fundamentally misguided if these models are not accompanied by evidence of neuropathology and neurobehavioral deficits that reflect corresponding abnormalities in blast-exposed humans. In this context, structure–function isomorphy represents a core principle for animal model comparison and experimental generalizability. Focus on blast (input) at the expense of brain (output) is unlikely to yield clinically relevant translational benefits of significance to blast TBI and sequelae in humans.

A number of additional translational issues merit consideration for TBI animal modeling. These include the nature of blast production and exposure, internal versus external placement and orientation of the animal subject relative to the blast tube, presence or absence of cervical and head immobilization [22],[39], use of thoracic protection, monitoring for blast-induced barotrauma and post-exposure apnea, anesthetic and analgesic effects with respect to cervical muscle tone and cerebral oxygenation, and consideration of ultrafast (for example, microsecond) timescales for biomechanical analyses. The need for multimodal analyses, biological–pathological correlation, and human clinicopathological validation is of paramount importance. How these and other TBI animal modeling issues are addressed within a specific experimental context will depend on the questions under investigation. Regardless, interpretation of the resulting experimental data is subject to the same validation criteria discussed above. Critical appraisal and expert consensus regarding these translational issues will go a long way towards fostering progress in the field.

## Conclusions

Although investigation of blast-related TBI is proceeding at an unprecedented pace, a number of unresolved issues hinder research and clinical progress. Impediments to effective clinical care include limited understanding of the detailed mechanisms and specific circumstances by which blast exposure leads to acute brain injury and chronic neurological sequelae, poor insight into how blast-related TBI may impact different brain structures leading to variable functional deficits (for example, frontal lobe and disconnection syndromes, chronic pain and major affective disorders, hypopituitarism and endocrinological disturbances), unknown influence of genetic risks and modulatory factors (for example, age, sex, medical history, prior trauma, comorbidities), and absence of validated diagnostic criteria, disease-linked biomarkers, and disease-modifying treatments that reflect understanding of the substrate pathobiology. Implementation of robust multidisciplinary research programs that utilize relevant blast TBI animal models validated by correspondence to human neuropathology and functional deficits will advance understanding of pathogenic mechanisms, accelerate translational progress, and benefit medical care for individuals affected by acute and chronic effects of blast-related neurotrauma.

## Abbreviations

CTE: Chronic traumatic encephalopathy

PTSD: Post-traumatic stress disorder

TBI: Traumatic brain injury

## Competing interests

The authors declare that they have no competing interests.

## Authors’ contributions

LEG wrote the review. ACM and PKS provided critical commentary and edited the review. The views expressed in this article are those of the authors and should not be construed as official positions of the Department of Veterans Affairs or the United States government. All authors read and approved the final manuscript.
